# Medical News and Miscellany

**Published:** 1895-04

**Authors:** 


### Medical News and Miscellany.

**The Small-pox** scare is about over; nobody hurt.

**Harvard** has abolished intercollegiate foot-ball. Sensible.

**Dr. J. P. Hendrick** has removed from Houston to Huntsville.

**Dr. A. C. DeLong** has removed from Gleam to Lexington, Texas.

**Dr. J. T. Carter** has removed from Warrenton to Walhalla, Texas.

**Doctor,** write to us about a new battery. We have them from $10 up, and will give you a bargain.

**The Peacock Chemical Company,** of St. Louis, Mo., have removed their office and laboratory to 112 and 114 North Second Street.

**Legislature** finally passed bill creating a School of Dentistry in the Medical Department of our University, and appropriated $15,000 therefor.

**In the** trial of Dr. E. Potthast, at Columbus, Texas, for the killing of Dr. Grace, at Weimar, Texas, the jury rendered a verdict of not guilty.

**Quarantine.**—The governor has issued his annual proclamation instituting quarantine against all ports south of latitude 25° N., to take effect May 1st, prox.

**Dr. James Kennedy,** of San Antonio, formerly Professor of

Pharmacy in the State School of Pharmacy, Galveston, died of Bright's disease, March 27th, age 32.

---

**Dr. M. K. Lott,** our esteemed friend, for four years the efficient quarantine inspector at Eagle Pass, was at the Capital this week, and honored the JOURNAL with a call.

---

**It is** to be hoped that a large delegation of Texans will attend the Baltimore meeting of the American Medical Association, on May 7-10. Every inducement is offered. See notice elsewhere.

---

**Dr. G. W. Wells,** of New York, associate medical director of the Manhattan Life Insurance Company, and editor of the *Medical Examiner*, was in Austin last week, and honored the JOURNAL'S office with a call.

---

**Dear Doctor.**—In making up your budget for expenses to Dallas, do not fail to put in the item, "$2 for subscription to the redback." There will be a paper in next issue, alone worth the price of a year's subscription.

---

**As we are** mailing out, there is going on in New Orleans a conference of Gulf State health officers, who are trying to devise a means of permitting the importation of fruit from tropical ports during the close season. It is a little risky, but it can be done.

---

**Remember** the American Medical Association meeting in Baltimore, May 7, 8, 9 and 10. This will no doubt be the largest meeting held for many years; the time of the year, reduced railroad rates, and the place of meeting, all favor a large attendance.

---

**Dr. Jno. Preston,** the long-time Superintendent of the N. W. Texas Insane Asylum, has located at Austin and engaged in general practice. The JOURNAL extends a cordial welcome to the doctor, and congratulates Austin upon this acquisition to her citizenship.

---

**Attention Curio-Seekers.** The JOURNAL is authorized to offer for sale a rare historical relic in the shape of a Mississippi and Alabama Railroad Bank Bill of March, 1837, in good preservation. This is a relic of the days of Mississippi's repudiation of her bonds.

**The Passing of the "American Lancet."**—With the issue of the *American Lancet* for the current month, Dr. Leartus Connor, after a service of nearly twenty-four years, retires from the editorial chair, and with his withdrawal the publication of the *Lancet* comes to end.

---

**Hypnotism in Court.**—A Missouri judge has recognized hypnotism as a factor in murder. A man committed a murder, and pleaded that he was induced to do it by another man. The murderer was acquitted, and the "other man" was convicted of the murder. Whither, oh, whither are we drifting?.

---

**Speaking** of large doses of morphine, we are promised for next issue Dr. J. F. Eaves' account of treating (with permang. pot.) the assassin who slew four persons at Millican recently. He attempted suicide by taking a dram of morphine. Dr. Eaves saved him, so he can now be hanged. Another triumph for science.

---

**The Buffalo Medical Journal** will celebrate its semi-centennial shortly, by increasing its reading pages from sixty-four to eighty, and making other improvements. The JOURNAL extends its congratulations to Brother Potter; but we want it distinctly understood that he has not been editing the *Journal* all these years. Long may he and the *Journal* live and prosper.

---

**For Ophthalmia Neonatorium,** a sure cure has been discovered, and that too, by a Texas legislator. When the bill to prevent blindness by compelling early attention to sore eyes in the new born, was before the legislature, an old fellow, calling himself "doctor," hooted at the idea, and said "a little mother's milk dropped in the baby's eye would cure it sure pop, every time." It killed the bill.

---

**His Occupation.**—Magnetic Healer Fannin, an individual well known in Texas, we are informed, has remained in Austin all winter, and has cultivated the rustic and populist element of the immortal 24th legislature to some purpose. To him is attributed, mostly, the defeat of the medical act, and he was heard to say that holding down medical legislation has been his occupation here ten years. He is the "vitopath" referred to in the senate discussion.

**Medical Editors,** publishers and business managers are cordially invited to attend the second annual meeting of the American Medical Publishers' Association, at the Eutaw House, Baltimore, Md., May 6, at 9:30 a. m. Subjects of vital importance will be discussed, and a profitable and pleasant session is anticipated.

LANDON B. EDWARDS, M. D.,
President, Richmond, Va.

CHARLES WOOD FASSETT,
Secretary, St. Joseph, Mo.

---

**The** *Columbus Medical Journal* has taken up the position of the TEXAS MEDICAL JOURNAL, the *Texas Sanitarian* and the *California Southern Practitioner* as to the insurance companies paying physicians for information when referred to by applicants for position as medical examiner, and the Editor, and Medical Director Curtis of the Equitable, are engaged in a bitter controversy. The *Journal* is right. It has ceased to be a question here, and the Austin physicians send in a bill for $5 every time a request is made for information about an applicant, and for our part we always get our money.

---

### The International Route.

Do you propose to attend the Baltimore meeting? If so, please remember the International Route and connections offer superior facilities.

Through sleepers, Laredo, San Antonio, Austin and Taylor to St. Louis and Chicago; San Antonio, Austin and Taylor to Memphis; Galveston, Houston and Palestine to St. Louis daily without change. Call on nearest I. & G. N. ticket agent for full information regarding rates, routes, etc.

D. J. PRICE, A. G. P. A.,
Palestine, Texas.

---

**Texas Graduates.**—In the *Memphis Medical Journal* we find an account of the Commencement Exercises of the Memphis Hospital Medical College, and amongst the graduates the names of the following Texas students. We will be pleased to receive from other colleges a list of Texas graduates for publication: A. J. Borden, Homer; A. A. Blasingame, Denman; J. E. Baldwin, Kleburg; W. M. Bowen, Navasota; J. W. Edwards, Mt. Pleasant; W. Y. Fowler, Valley Springs; A. J. Halbrook, Marietta; R. H. Lasator, Haughts Store; F. V. McKnight, Black Oak; J.

A. T. Page, Paige; S. S. Robinson, Arthur City; W. C. Shaw, Kaufman; W. C. Taylor, Franklin; J. M. Wolfe, Jacobi; J; S. Zvesper, Ammansville.

---

**Journal Pi(e).**--The *Journal of the American Medical Association* came near losing its plant by fire, last week; a fire broke out in the printing office, and but for prompt discovery and prompt action, the entire concern would have been destroyed. As it was, the composition was "pied." We don't know whether the entire plant was pied or not, nor whether in fact it was a pie plant. It may have been a rhubarb plant (aye, there's the rhub). The office was deluged by the fire engines, but Editor Hamilton's spirits was not in the least dampened (had it in the closet, may be) by having cold water thrown over his work, as he triumphantly announces that the *Journal* came out on time, notwithstanding. Hamilton is the "hot stuff," and a little fire doesn't scare him worth mentioning.

---

**Louisville Doctors have a Street Fight.**—The *Courier-Journal* of April 4th, contains a lengthy account of a street fight between Dr. D. S. Reynolds, a well-known occulist of that city, and Dr. Ed Richardson. It seems that Reynolds got the "best" of it, if there was any "best" to so disgraceful an affair; neither was hurt, barring a slight scalp wound on the back of Richardson's head, where it came in contact with the pavement. The account says that it is the result of an old feud; that Richardson confesses to have written several years ago a defamatory letter about Reynolds to "an oculist in Texas," which letter was published in Louisville, and claims that he struck Reynolds for looking at him as they passed on the street; while Reynolds says he knocked Richardson down,—held him down, but forbore to punish him,—for striking at him. "Doctors will differ," but they shouldn't fight.

---

**Patent Communion Service.**—A Michigan clergyman thinks he has solved the individual cup problem. The Rev. Charles E. Lee, of the Second Baptist Church, Grand Rapids, Mich., has invented an individual church communion service and has had it patented. It consists of a pyramid of three disks attached to a central standard. The disks have apertures into which seventy-five tiny cups shaped like grapes repose, each holding a spoonful of wine. At the top of the standard is a ring and swivel. A

long handle with a hook at the end is used in passing the standard. The hook fits in the ring at the top, and by turning the disks around, the filled cups are always easily reached. As rapidly as used the cups are inverted, and there is an attachment to catch drippings. The disks and standard are made of any metal desired, and are susceptible of elaborate ornamentations. The grape cups may be of silver, gold, or china, according as a church may desire.—*N. Y. Med. Record.*

---

**Editor Hamilton,** of the *Journal of the American Medical Association,* says: "We have received a great many photographs of members of the Association, but a very small percentage of the membership," and calls on all hands to send in their phiz by *return mail.* What's he going to do with them? Good Lordy! not going to print them, I hope! What have they been cured of? and was it Hood's, or Celery Compound? It is to be hoped they are not to be attached to papers and sent out with reprints. That thing is getting very common; in fact, more common than proper. Br. Bernays sent out one recently. It is not in good taste. We always think of Douglas' $3 shoe, and Beeman's chewing gum. Do those fellows think they are pretty? We don't; but there' no accounting for taste. It might be justified on the grounds of "practical teaching," like old Squeers, the Yorkshire school-master; he made Smike spell "horse," and then go and curry his horse, so as to impress it on his mind.

---

**An American Milk Yarn.**—E. Marsh, of Mineral Township, in America, comes to the front with a story that is both wonderful and unique, but true in every detail. About five weeks ago a Durham-Alderney cow, aged two years, gave birth to a calf, and they began to milk the cow, but were thunderstruck when they discovered that its milk was black. The calf, however, thrived upon the milk, and last week Mrs. Marsh, having overcome her prejudices, decided to try some of the milk. It tasted the same as other milk, only it was much richer, and by leaving a crock of it to set four hours, nearly two inches of cream, a little lighter in color than the milk, would rise to the top. Two gallons of the cream were churned and four pounds of butter were secured. The butter was examined by a chemist, who pronounced it perfect butter only in color, and gave the reason for the color, something yet unknown to science in the blood of the animal. The butter much resembles coal tar, and has a delicious taste. People are coming from far and near to see the freak, and Mr. Marsh has been offered big sums for the cow. He has decided not to sell her, however, in the hope that he can raise some more stock of the same kind. A roll of butter will shortly be sent to Franklin and placed on exhibition. The milk makes fairly good ink and the cream might be used for printers' ink.—*Food and Sanitation.*

---

**Grape Seeds in a Healthy Appendix.**—Dr. McBurney presented a vermiform appendix containing two grape seeds, removed under the following circumstances: He was operating upon a patient for abdominal tumor; the healthy-looking appendix thrust into view, and on feeling of it he appreciated two concretions. Thinking it wise to act in time, he removed it, and the bodies which had been felt were found to be two grape seeds. The notion had been very prevalent that grape seeds were dangerous on account of their liability to lodge in the appendix and set up inflammation. Dr. McBurney had received letters regarding this matter from various parts of the country, a recent one being from a large horticulturist in the West, who said he would be greatly indebted to him if he could state that grapes were not dangerous. Dr. McBurney had always been able to reply that he thought grapes were harmless, since he had never seen a grape seed in the concretions contained within the appendix. It had so happened that the first exception to this experience was in a case in which he had incidentally removed the healthy appendix and found within it two grape seeds.

Dr. Polk remarked that he had seen grape seeds cause appendicitis in but one case.

Dr. Biggs made some remarks upon the work of th Board of Health in producing diphtheria antitoxin for use in the city.—*N. Y. Medical Record.*

---

**Diphtheritic Conjunctivitis Treated by Serotherapy.**—Dr. Jessup recently communicated a report to the Royal Ophthalmological Society of two cases of diphtheritic conjunctivitis treated by Klein's antitoxin. The first case was that of a boy, aged 19 months, with false membrane on the conjunctivæ of both eyelids of the left eye, with a patch on the uvula, swelling of the subparotid lymph glands and albuminuria. Three injections were made; the total quantity of the antitoxin used was three grams. The membrane disappeared in five days without leaving any trace on the conjunctivæ, though the only local application used was distilled water. In the second patient, also a boy aged 8 months, there were membranes on the palpebral conjunctivæ of both eyes, enlarged glands and a muco-purulent discharge from the nostrils. Two grams of the antitoxin were used in two injections and the false membrane disappeared in four days. Hayward examined the membranes in both cases and discovered large quantities of the Löffler bacillus. In his opinion the cure was certainly due to the antitoxin, because these cases are generally accompanied with purulent ophthalmia and ocular lesions which are very slow in healing. Coppey, of Brussels, has also reported a case in a little girl of 1 year, with severe ocular diphtheria cured in four days after one injection of Behring's serum. —*Journal A. M. A.*

---

**Just after the ignominious failure** of the Wooten bill some of the medical gentlemen of San Antonio, sent up a bill to regulate the practice, a very thorough bill, a bill which requires examination, the only test of qualification, and it was introduced into the house, and duly referred to the committee on public health. Drs. J. V. Spring, B. F. Kingsley and F. M. Hick, of San Antonio, and Dr. Bennett, of Austin, appeared before the committee and presented the case. The chairman, a Mr. Bumpass, asked Dr. Spring "if a man owns a cure for cancer, a man not educated in medicine, but who can cure cancer by local application, would your bill exclude him?" Dr. Spring replied that examination upon the various branches of medicine was the test, and if his man was ignorant, as admitted, and could not pass examination, of course he would be rejected. Mr. B, then—we are informed—pointed to scars on his own face and claimed that he had been cured of cancer by this man and his salve, when the doctors had failed, and for his part, he would insist on allowing such men to practice.

It is hopeless to expect ever to educate such a blockhead up to a comprehension of the situation. Well,—what do you expect of men who can leave home and business and come here and serve the State as legislators at $2 a day? After the first sixty days—during which time they get $5 per day—the pay is $2, and the way they hold on, away up towards May, is proof conclusive that the pay is the inducement. So long as Texas pays her legislators laborers' wages we may expect just such displays of ignorance. There are intelligent gentleman, and many of them, who come here at heavy loss; but they are in the minority, it seems.

---

**A chance to take a post-graduate course** in Baltimore, Philadelphia or New York, and attend the big meeting of the American Medical Association, all on one trip, and reduced railroad rates, is now open to the physicians of Texas. This is an unusual opportunity, and we believe that not a few of our readers will take advantage of it. The meeting promises to be one of the largest ever held, and the social features will surpass anything that has preceeded it. Various entertainments in the city of Baltimore, excursions to Washington, New York, Old Point Comfort and Gettysburg are among the good things promised. The trip to Baltimore will be a most pleasant one. Good train service, the best of accommodations and quick time are assured. If a party of Texas physicians can be made up to meet in Texarkana on May 4th, they can reach St. Louis by the morning of May 5th, where they will join the delegation from that city, and travel with them over the Big 4 and the C. & O. roads to Washington, thence to Baltimore. The I. & G. N. and the Texas & Pacific roads will have tickets on sale in ample time to reach Texarkana by the 4th, and the Iron Mountain train will arrive in St. Louis on the 5th, and make close connection with the Big 4 train which will carry the St. Louis party. The route through Arkansas and Missouri over the Iron Mountain, from St. Louis to Indianapolis and Cincinnati over the Big 4, and from Cincinnati through West Virginia and Virginia and to Washington over the Chesapeake and Ohio, is one of the very best and most picturesque that could have been selected. The scenery is magnificent throughout almost the entire route, and that in the Alleghany and Blue Ridge mountain ranges is unsurpassed any where.

Parties from Galveston, Houston, San Antonio, Austin, and intermediate points, should start on the night of May 3d, and those from Dallas and Fort Worth on the morning of the 4th, reaching Texarkana in time for the Iron Mountain Cannon-Ball train, which leaves Texarkana for St. Louis at 3 p. m. on the 4th.

See that your tickets read via the Iron Mountain, Big 4, and Chesapeake & Ohio railways.

We hope to see a full attendance from Texas, and we can assure everyone of a pleasant and profitable trip.

**Large Doses of Morphine.**—W. A. Clark, M. D., contributes the following to the New York *Medical Record:* "The following is the history of a case in which I failed to obtain any narcotic effect from some quite large hypodermic injections of sulphate of morphine.

"Mrs. M——, aged sixty-seven, widow, no children, laundress. About two years ago was given several hypodermic injections of morphine for severe abdominal pain. Powders containing the drug were soon substituted and gradually increased, until before her death she was consuming twelve grains of morphine daily.

"January 16th.—Aneurism of abdominal aorta diagnosed, and being in considerable pain, she was given the following:

℞ Morph. sulph .................... gr. xxx
Aqua ........................... ℥iv
Sig: Teaspoonful every quarter hour until relieved.

"The hospital steward being called during the night, gave the following quantities at quarter-hour intervals: One-quarter grain, hypodermically; four grains by the mouth; two grains, hypodermically; three grains respectively; no result.

"January 17th—Remedy repeated as before. I was called about 7:30 p. m., and found her in intense pain. I gave the following quantities hypodermically, at quarter-hour intervals, commencing at 8 p. m.:

"Three grains, five grains, seven grains, in two injections, eight grains in two injections, six grains; no effect. Eight grains by the mouth was then given until 11 p. m., when the stock on hand gave out, making a total of eighty-three grains consumed in three hours, with no appreciable effect. The sulphate of morphine had been prepared by a reliable firm, one-quarter grain from the same bottle giving a full effect in another patient. I might add that she had been consuming, daily, eight ounces each of whisky and port wine, and a quart bottle of beer, until about two weeks before her death, which occurred January 21, 1895.

"At the post-mortem examination no aneurism was found. Under the right kidney half of a hair-pin old, and corroded was discovered, with considerable localized peritonitis and old adhesions."

---

**Another Texas Journal.**—We have received Vol. 1, No. 1, of *The Hygeia*, and welcome it to our table and exchange list. It is a neat little journal of twenty-eight pages—in a pretty pink cover, issued by Drs. T. J. Bell & F. G. Kirkscey, at Tyler, Texas. Dr. Bell is one of the best known, and deservedly popular physicians in Texas, and stands high in the profession, having been first vice-president of the Texas State Medical Association, and has several times presided as chairman of important sections. His ambition to give East Texas a publication in the interest of "Hygiene and Curative Medicine," and incidentally devoted to organizing the medical men of the East, is commendable, and should he fail of realizing his fondest hopes, it will not be for lack of merit or effort on his part. We wish the fledgling abundant success, and its worthy senior editor,—our friend Bell,—especially, a long career of usefulness in the field of journalism.

The editors launch their little venture with a classical salutatory,--pay tribute to the Gods of medicine of old time, touch upon the benefits and blessings of hygiene, and in closing (with an eye to business) simulate the immortal Wegg--and drop into poetry; thus:

"So then 'Hygeia' is afloat;
And with sails well trimmed, and machinery all new,
We are encouraged to believe we will carry her through.
We only ask our friends to give us a pull,
And not kick us, and cuff us, and treat us cool;
That when our barque is safe on the other side
East Texas may boast with pardonable pride
Of the little 'Hygeia,' the Gem of the East,--
The land of milk and honey—the best in the world!
Then, give us your aid,
Friends,—we are not begging, nor want to be paid,
For what we don't earn,
Or give value received to you in return!"

We had not before suspected that our friend Bell was a poet; that to his other gifts of mind was added the "divine afflatus;" or, did the other editor thus eloquently and touchingly fling *Hygeia's* "banner to the breeze?" May favoring winds waft her to a safe haven; and we trust no one will "treat her cool."

---

**Condensed Milk.**—It is decidedly refreshing in these days of sanitary scares and sensations to meet with such a common-sense and encouraging utterance as that of Prof. Albert Leeds, chemist of the Stevens Institute of Technology, on the subject of condensed milk. In consequence of sundry sensational newspaper items on the subject, published last summer, the professor was requested by the New Jersey State Board of Health, and by some local boards, to investigate the various brands—some fifteen in all—of condensed milk in the market, and his report is now made public in the current number of the *American Journal of the Medical Sciences.* As was to be expected, some of the specimens were condemned—more particularly those which had undergone changes due to specific bacterial ferments getting into the milk before the process of condensation. But instead of making these the theme of a general hysterical condemnation of all condensed milk, the analyst sensibly points out that the interests of the public coincide with those of the manufacturers in most respects, and that, apart from the propriety of its use for infant nutrition, much can be said in praise of the great value of condensed milk as a manufactured product. He says that it would be a great loss to the canners were they to use or purchase watered milk, since the chief expense of the condensation is in the evaporation of the water; and dairymen dishonest enough to water milk are those most likely to have impure wells, improper food and unclean surroundings. Not from added water, but from water used in washing utensils is danger to be dreaded, and the same remark is true of the original milk itself. It is out of the question, Professor Leeds says, for a canner to employ a bacteriologist to examine the milk used, and the hardening and putrefactive changes which the condensed milk sometimes undergoes are usually due to failures in cleanliness, etc., at the dairy. Later on the canner will participate with the public in the great benefits of a rigid system of inspection that will, first of all, begin with the dairy and the cattle themselves. The *Journal* is aware that, as a result of municipal milk inspection in many large cities, the dealers themselves are the most efficient agents in keeping the producer up to the mark. They not alone have frequent analysis of their supplies made by the municipal analysts, but many dealers employ appliances for milk analysis in their own depots, and are thus enabled to detect promptly the grosser forms of adulteration and to locate the responsibility.—*Journal A. M. A.*

---

**The extreme of parsimony** was manifested in the Texas legislature last week when an amendment to the appropriation bill was introduced (and is now in great danger of passing), cutting off the provision supply of Asylum Superintendents and Assistant Superintendents. The law gives the Superintendents $2500 a year and their boarding and rooms; it requires them to "live at the asylum," and to give their whole time to the institution. The 24th has reduced the pay to $2000, and now seeks to compel them to either pay their board to the State, or to go elsewhere to board, or to furnish themselves. This is simply infamous; it is a great wrong to the unfortunates, for no qualified physician, capable of ministering to their needs, can be found to take a superintendency at $166 a month and find himself and family. That the bill will, if it pass, cripple the service; compel those now in charge to resign, is almost certain. The JOURNAL sincerely hopes that a wiser policy may prevail.

---

**A Dangerous Thing.**—Hostess: Mr. Dinerout, are you not going to eat your strawberries, they are the first of the season?

Mr. Dinerout (a chronic boarder): Thanks; not if I know myself; *they will spoil my appetite for prunes*."

---

### The Antitoxine Treatment of Diphtheria.

There are signs that we shall soon be prepared in this country to give this treatment all the trial that may be needed to demonstrate either its efficiency or its comparative worthlessness, for the sources of abundant supplies of the antitoxine are fast multiplying. Among recent additions to these sources is the Massachusetts board of health. The *Boston Medical and Surgical Journal* for March 28th, publishes an official statement by the secretary of the board that it has prepared a supply of antitoxine for the benefit, primarily, of such communities in the State as find it difficult or impracticable for any reason to supply themselves with the new agent from reliable sources. We are glad to see the following passage in the statement: "The board *does not propose to offer it for sale*, but its gratuitous distribution will be under strict conditions designed to prevent abuse and waste and to obtain the most beneficial fruits. Each bottle is marked with a number and the date of the preparation of its contents. No antitoxine will be issued except upon a pledge that a full statement of the observed effects of its use will be returned to the board at the termination of the case. In all instances possible a bacterial diagnosis will be insisted upon." It will be seen that the Massachusetts board does not seek to attach a commercial tail to its operations and therein it shows due respect for the rights of citizens of the State, while in no wise neglecting anything that an official sanitary board may properly undertake in the way of facilitating the amplest trial of the new treatment.

Doubtless it is yet too early to declare that the efficiency of the antitoxine treatment has been demonstrated, but it must be said that the prospect of such a demonstration is apparent. Whenever any inquiry of this sort is before the profession, and especially when it is before the public, as in this instance, there are never wanting those who set their faces with the fixity of fanaticism against the measure, can see no good in anything so novel and radical, and seize upon every misadventure in its employment to confirm and justify their opposition. Such a misadventure has recently happened in Brooklyn—namely, the speedy death of a diphtheria patient after receiving an injection of the Behring serum. The death has not at the time of our going to press been explained satisfactorily, but we feel sure that no blame can be attached either to the physician who gave the injection, or to the firm that imported the serum. The unfortunate occurrence enforces, however, the need of minute caution.—*Ed. in N. Y. Med. Journal* April 6.

[Since the above was printed we have received a circular issued by W. H. Park, M. D., Assistant Director of Hospital Bacteriological Laboratory of the New York City Board of Health, accompanied by a certificate from E. H. Wilson, M. D., Chief Bacteriologist of the Brooklyn Health Department, to the effect that two vials of the antitoxine of the same lot as that used by Dr. Kortright in the case of Bertha Valentine, whose violent death followed in ten minutes after the injection, was submitted to bacteriological tests, and were found to be absolutely free from living germs of any kind, and Dr. Park says further, the unfortunate result * * * can not be attributed in any way to the antitoxine which was employed.

Dr. Wilson says: "Speculative theories may be advanced as to the cause of death in this case, the true cause not having yet been determined, but the above experiment [omitted here] demonstrates that the cause was not inherent in the antitoxine." —Ed.]

---

### Hydrophobia Inoculation.

Dr. A. Lagorio, Director of the Pasteur Institute, of Chicago, furnishes the following summary of the preventive inoculation against hydrophobia attained at that institution since its inauguration, July 2, 1890:

From July 2 to December 31, 1890, persons treated, 37.

From January 1 to December 31, 1891, persons treated, 62.

From January 1 to December 31, 1892, persons treated, 101.

From January 1 to December 31, 1893, persons treated, 107.

From January 1 to December 31, 1894, persons treated, 59.

Total, 366.

341 persons were bitten by dogs, 9 by horses, 7 by cats, 5 by skunks, 2 by wolves, 1 by a mule and 1 by a pig.

195 persons received severe and multiple bites on the hands and wrists, 47 on the head and face, 47 on the arms and shoulders, and 77 on the legs and thighs.

Following the role of Pasteur the patients treated have been classified into three categories:

First—Persons bitten by animals recognized and ascertained to be rabid by the control experiment made in the laboratory or by the death of other persons or animals bitten by the same animal. Of this category 123 were treated.

Second—Persons bitten by animals recognized to be rabid by the symptoms of the disease shown during life. Of this category 160 were treated.

Third—Persons bitten by animals strongly suspected to be rabid. Of this category 83 were treated.

The number of persons bitten by rabid animals, prior to the Pasteur anti-rabic treatment, was as follows:

Eighty-eight per cent for the bites of head and face, sixty-seven per cent for the bites of the hands, and twenty to thirty per cent for the bites of the limbs and trunk. We feel, therefore, pleased at the results attained at this Institute, as only two deaths have been reported out of the 366 patients treated; thus giving a mortality of 0.54 per cent.

Besides the above 372 persons applied for treatment, but were sent back, as it was recognized, either by the control experiments or by history of the case, that the animal was not rabid; their wounds, however, were treated free of charge.

The Institute will thankfully acknowledge any assistance or donations from private individuals or institutions contributed for the relief of the poor.

